# Human serum from SARS-CoV-2-vaccinated and COVID-19 patients shows reduced binding to the RBD of SARS-CoV-2 Omicron variant

**DOI:** 10.1186/s12916-022-02312-5

**Published:** 2022-03-03

**Authors:** Maren Schubert, Federico Bertoglio, Stephan Steinke, Philip Alexander Heine, Mario Alberto Ynga-Durand, Henrike Maass, Josè Camilla Sammartino, Irene Cassaniti, Fanglei Zuo, Likun Du, Janin Korn, Marko Milošević, Esther Veronika Wenzel, Fran Krstanović, Saskia Polten, Marina Pribanić-Matešić, Ilija Brizić, Fausto Baldanti, Lennart Hammarström, Stefan Dübel, Alan Šustić, Harold Marcotte, Monika Strengert, Alen Protić, Antonio Piralla, Qiang Pan-Hammarström, Luka Čičin-Šain, Michael Hust

**Affiliations:** 1 Technische Universität Braunschweig, Institut für Biochemie, Biotechnologie und Bioinformatik, Abteilung Biotechnologie, Spielmannstr. 7, 38106 Braunschweig, Germany; 2 Helmholtz Centre for Infection Research, Department of Viral Immunology, Inhoffenstr. 7, 38124 Braunschweig, Germany; 3 Microbiology and Virology Department, Fondazione IRCCS Policlinico San Matteo, 27100 Pavia, Italy; 4 Department of Biosciences and Nutrition, Karolinska Institutet, Huddinge, Sweden; 5 Abcalis GmbH, Science Campus Braunschweig-Süd, Inhoffenstr. 7, 38124 Braunschweig, Germany; 6 Department of Anesthesiology, Reanimation, Intensive Care and Emergency Medicine, Faculty of Medicine, University of Rijeka, Rijeka, Croatia; 7 Center for Proteomics, Faculty of Medicine, University of Rijeka, Rijeka, Croatia; 8 Department of Clinical, Surgical, Diagnostic and Paediatric Sciences, University of Pavia, Pavia, Italy; 9 Department of Epidemiology, Helmholtz Centre for Infection Research, Inhoffenstr. 7, 38124 Braunschweig, Germany; 10 Centre for Individualised Infection Medicine (CIIM), a joint venture of Helmholtz Centre for Infection Research and Medical School Hannover, Hannover, Germany

**Keywords:** SARS-CoV-2, Omicron variant (B.1.1.529), Delta variant (B.1.617.2), Beta variant (B.1.351), Vaccination, Antibody titer, COVID-19, Virus neutralization, Human angiotensin-converting enzyme-2 receptor (ACE2), Receptor-binding domain (RBD)

## Abstract

**Background:**

The COVID-19 pandemic is caused by the betacoronavirus SARS-CoV-2. In November 2021, the Omicron variant was discovered and immediately classified as a variant of concern (VOC), since it shows substantially more mutations in the spike protein than any previous variant, especially in the receptor-binding domain (RBD). We analyzed the binding of the Omicron RBD to the human angiotensin-converting enzyme-2 receptor (ACE2) and the ability of human sera from COVID-19 patients or vaccinees in comparison to Wuhan, Beta, or Delta RBD variants.

**Methods:**

All RBDs were produced in insect cells. RBD binding to ACE2 was analyzed by ELISA and microscale thermophoresis (MST). Similarly, sera from 27 COVID-19 patients, 81 vaccinated individuals, and 34 booster recipients were titrated by ELISA on RBDs from the original Wuhan strain, Beta, Delta, and Omicron VOCs. In addition, the neutralization efficacy of authentic SARS-CoV-2 wild type (D614G), Delta, and Omicron by sera from 2× or 3× BNT162b2-vaccinated persons was analyzed.

**Results:**

Surprisingly, the Omicron RBD showed a somewhat weaker binding to ACE2 compared to Beta and Delta, arguing that improved ACE2 binding is not a likely driver of Omicron evolution. Serum antibody titers were significantly lower against Omicron RBD compared to the original Wuhan strain. A 2.6× reduction in Omicron RBD binding was observed for serum of 2× BNT162b2-vaccinated persons. Neutralization of Omicron SARS-CoV-2 was completely diminished in our setup.

**Conclusion:**

These results indicate an immune escape focused on neutralizing antibodies. Nevertheless, a boost vaccination increased the level of anti-RBD antibodies against Omicron, and neutralization of authentic Omicron SARS-CoV-2 was at least partially restored. This study adds evidence that current vaccination protocols may be less efficient against the Omicron variant.

**Supplementary Information:**

The online version contains supplementary material available at 10.1186/s12916-022-02312-5.

## Background

SARS-CoV-2 is the etiological agent of the severe pneumonia COVID-19 (coronavirus disease 2019) [1, 2]. A new variant B.1.1.529 of the betacoranavirus SARS-CoV-2 was identified in late November 2021 and has rapidly been classified as a variant of concern (VOC) by the WHO and named Omicron [3]. The Omicron variant shows a high number of mutations in the SARS-CoV-2 spike protein in comparison to the previously described VOCs Alpha [4], Beta (B.1.351) [5], Gamma (P.1) [6], and the currently dominating Delta variant (B.1.617.2) [7]. The first sequenced Omicron variant (GISAID accession ID EPI_ISL_6913995, collection date 2021-11-08, South Africa) contains a total of 36 mutations compared to the original Wuhan strain and includes 29 amino acid (aa) changes, six aa deletions, and one aa insertion. Fifteen of these mutations are concentrated in the N-terminal receptor-binding domain (RBD) of the spike protein which binds to the human zinc peptidase angiotensin-converting enzyme 2 (ACE2) for cell entry [8, 9].

Importantly, the RBD is targeted by more than 90% of the neutralizing serum antibodies, making it the most relevant target for SARS-CoV-2 neutralization [10, 11]. Consequently, the majority of therapeutic antibodies for the treatment of COVID-19 are designed to interact with this part of the SARS-CoV-2 spike protein [12, 13]. The abundant mutations in spike might indicate that the Omicron variant may bind with a different affinity to the ACE2 receptor, therefore may be altering its cell entry characteristics. Simultaneously, the mutations may help the virus to escape the immune recognition by antibodies, facilitating viral spread in a seropositive population.

While initial studies have shown a severe reduction in serum neutralizing capacity of vaccinated and convalescent patients against the Omicron variant [14–16], it is unclear to which extent the RBD domain mutations contribute to this loss in neutralization activity. Additionally, while several mutations present in Omicron are computationally predicted to increase ACE2 binding affinity, others are predicted to reduce its affinity [17].

The aim of this study is the analysis of the new Omicron RBD and unravel why the Omicron variant is displacing other variants. Therefore, the binding of ACE2 to the new Omicron RBD was determined in comparison to the original Wuhan strain and the Beta and Delta variants by two different techniques. Moreover, we tested the binding of human sera from COVID-19 hospitalized patients or vaccinated persons with 2× BNT162b2, 1× Ad26.COV2.S, or 2× mRNA1273 vaccines, as well as boost vaccinated persons, to the RBD of the original Wuhan strain, the Beta, the Delta, and the Omicron VOC. Furthermore, the neutralization efficacy of sera of 2× BNT161b2 and boost vaccinees was analyzed using the authentic SARS-CoV-2 virus.

## Methods

### Serum samples

Blood samples were obtained from non-vaccinated, intensive care unit (ICU) patients with severe symptoms from the second (pre-Alpha) and third (Alpha variant) pandemic wave in Croatia (Rijeka, sampling December 2020 to April 2021) and Italy (Pavia, sampling March 2020 to February 2021) or from vaccinated people in Germany (Braunschweig, sampling June 2021 to December 2021), Sweden (Stockholm, May 2021 to November 2021), and Italy (Pavia, February 2021 to January 2022) as indicated. While all voluntary donors were informed about the project and gave their consent for the study, consent requirement was waived by the ethical committee in Rijeka for patients in intensive care where sampling was a part of routine diagnostics. Ten out of the 17 patients of the Croatian cohort died by COVID-19 infection. The sampling was performed in accordance with the Declaration of Helsinki. The donors included adults of both sexes. The first WHO International Standard for anti-SARS-CoV-2 immunoglobulin (NIBSC code: 20/136) was used as positive control serum, and pre-pandemic negative control sera were provided by the LADR Braunschweig and did not bind to any RBD variant (data not shown). Approval was given from the ethical committee of the Technische Universität Braunschweig (Ethik-Kommission der Fakultät 2 der TU Braunschweig, approval number FV-2020-02). The study in Croatia was approved by the Ethics committee of the Rijeka Clinical Hospital Center (2170-29-02/1-20-2). The study in Italy was performed under the approval of the Institutional Review Board of Policlinico San Matteo (protocol number P_20200029440). The study in Sweden was approved by the ethics committee in Stockholm (Dnr 2020-02646).

Details about study participants are shown in Table 1.

**Table 1:** Used human serum samples in this study

		***n (female/male)***	***Mean age (range)***	***Time point of sampling***
Patients	Severe symptoms, hospitalized (ICU), unvaccinated	27 (7/20)	65 (39–86)	7–25 days after symptom onset (mean 12 days)
Vaccinated persons	2×BNT162b2 (Corminaty, BioNTech-Pfizer)	69 (40/29)	42 (23–66)	7–54 days after 2nd dose (mean 22 days)
	2×mRNA-1273 (Spikevax, Moderna)	6 (2 /4)	38 (19–70)	5–55 days after 2nd dose (mean 26 days)
	1×Ad26.COV2.S (Janssen COVID-19 vaccine, Johnson&Johnson)	6 (2/4)	35 (24–40)	14–33 days after 1st dose (mean 26 days)
	2× BNT162b2 or 1×Ad26.COV2.S boosted by 1× BNT162b2 (or mRNA-1273)	34 (20/14)	39 (24–66)	5–49 days after 3rd/2nd dose (mean 19 days)

### Construction of the expression vectors

All sequences of the RBD variants (319-541 aa of GenBank: MN908947) were inserted in a *Nco*I/*Not*I compatible variant of the OpiE2 expression vector [18] containing an N-terminal signal peptide of the mouse Ig heavy chain and a C-terminal 6xHis-tag. Single-point mutations to generate the Beta and Delta variants of RBD were inserted into the original Wuhan strain through site-directed mutagenesis using overlapping primers according to Zheng et al. [19] with slight modifications: S7 fusion polymerase (Mobidiag) with the provided GC buffer and 3% dimethyl sulfoxide was used for the amplification reaction. The RBD Omicron variant was ordered as GeneString from GeneArt (Thermo Fisher) according to EPI_ISL_6590608 (partial RBD Sanger sequencing from Hong Kong), EPI_ISL_6640916, EPI_ISL_6640919, and EPI_ISL_6640917 including Q493K which was corrected later to Q493R. Table 2 gives an overview about the used variants.

**Table 2:** RBD variants used in this study (319-541 of GenBank: MN908947)

RBD wt	Original Wuhan	-
RBD Beta	B.1.351	K417N, E484K, N501Y
RBD Delta	B.1.617.2	L452R, T478K
RBD Omicron	B.1.1.529	G339D, S371L, S373P, S375F, K417N, N440K, G446S, S477N, T478K, E484A, Q493K, G496S, Q498R, N501Y, Y505H

### Expression and purification of the RBD variants

The different RBD variants were produced in the baculovirus-free High Five cell system [20] and purified as described before [21]. Briefly, High Five cells (Thermo Fisher Scientific) were cultivated at 27°C, 110–115 rpm in EX-CELL 405 media (Sigma Aldrich) at a cell density between 0.3 and 5.5 × 10^6^ cells/mL. On the day of transfection, cells were centrifuged and resuspended in fresh media to a density of 4 × 10^6^ cells/mL before 4 μg expression plasmid/mL and 16 μg/mL of linear PEI 40 kDa (Polysciences) was pipetted directly into the cell suspension. After 4–24 h, cells were supplemented with fresh media to dilute the cells ~1 × 10^6^ cells/mL, and 48 h after transfection, culture volume was doubled. Cell supernatant was harvested 4 to 5 days after transfection by a two-step centrifugation (4 min at 180 ×*g* and 20 min at >3500 ×*g*) and then 0.2 μm filtered for purification. Immobilized metal ion affinity chromatography (IMAC) His tag purification of insect cell supernatant was performed with a HisTrap excel column (Cytiva) on Äkta system (Cytiva) according to the manufacturer’s manual. In a second step, the RBD domains were further purified by size exclusion chromatography (SEC) by 16/600 Superdex 200 kDa pg column (Cytiva).

### Expression and purification of ACE2-hFc

The extracellular domain of ACE2 receptor (GenBank NM_021804.3) was produced in pCSE2.6-hFc expression vector in Expi293F cells (Thermo Fisher Scientific) as described before [22]. In brief, Expi293F cells were cultivated at 37°C, 110 rpm, and 5% CO_2_ in Gibco FreeStyle F17 expression media (Thermo Fisher Scientific) supplemented with 8 mM Glutamine and 0.1% Pluronic F68 (PAN Biotech). For transfection, 1 μg DNA and 5 μg of 40 kDa PEI (Polysciences) per mL transfection volume were diluted separately in 5 transfection volumes and then mixed for the formation of complexes (20–30 min). Afterwards, PEI:DNA complexes were added to 1.5–2 × 10^6^ cells/mL. Forty-eight hours later, the culture volume was doubled by feeding HyClone SFM4Transfx-293 media (GE Healthcare) supplemented with 8 mM Glutamine and HyClone Boost 6 supplement (GE Healthcare) with 10% of the end volume. One week after transfection, the supernatant was harvested by 15 min centrifugation at 1500 ×*g*. Purification was performed on a 1-mL HiTrap Fibro PrismA (Cytiva) column on Äkta go (Cytiva) according to the manufacturer’s manual.

### ACE2 binding to RBD analyzed by titration ELISA

ACE2 binding to the produced RBD variant antigens was analyzed in ELISA in triplicates where 300 ng RBD per well was immobilized on a Costar High binding 96-well plate (Corning, Costar) at RT for 1 h. Next, the wells were blocked by 330 μL 2% MPBST (2% (w/v) milk powder in PBS; 0.05% Tween20) for 1 h at RT and then washed 3 times with H_2_O and 0.05% Tween20 (BioTek Instruments, EL405). ACE2-hFc was titrated from 0.01 mg/mL down to 1 ng/mL and incubated 1 h at RT prior to another 3× times washing step. Detection was performed by goat-anti-hIgG(Fc) conjugated with HRP (1:70,000, A0170, Sigma) and visualized with tetramethylbenzidine (TMB) substrate (20 parts TMB solution A (30 mM potassium citrate; 1% (w/v) citric acid (pH 4.1)) and 1 part TMB solution B (10 mM TMB; 10% (v/v) acetone; 90% (v/v) ethanol; 80 mM H_2_O_2_ (30%)) were mixed). After addition of 1 N H_2_SO_4_ to stop the reaction, absorbance at 450 nm with a 620-nm reference wavelength was measured in an ELISA plate reader (BioTek Instruments, Epoch). EC_50_ were calculated with OriginPro Version 9.1, fitting to a five-parameter logistic curve.

### Affinity measurement by microscale thermophoresis

The affinity measurements were performed as described before [23]. In brief, ACE2-hFc was labeled by the Protein Labeling Kit RED-NHS 2nd Generation (NanoTemper) according to the manufacturer’s protocol. A degree of labeling (DOL) of < 3 was achieved and 10 nM of the labeled ACE2-hFc was applied in the measurements. Titration of the RBD variants was done by a Precision XS microplate sample processor (BioTek) in 384-well plates. Measurement was performed in Monolith (Nanotemper) using Monolith NT. Automated Capillary Chips (NanoTemper). The Excitation-Power was set to 40% and MST-Power to medium. The timeframe of 0.5 s up to 1.5 s was chosen to analyze the data by the MO Affinity Analysis software (NanoTemper) by Hill fit. For all RBD variants, a signal response above 18 and a signal to noise above 40 was obtained.

### Serum titration ELISA

For titration ELISA, sera were diluted 1:100 to 1: 9.19 × 10^7^ in 384-well microtiter plates (Greiner Bio-One) coated with 30 ng/well of the respective RBD variant. In addition, all sera were also tested at the lowest dilution (1:100) for determination of unspecific cross-reactivity on Expi293F cell lysate (30 ng/well), BSA (30 ng/well), and lysozyme (30 ng/well). IgGs in the sera were detected using goat-anti-hIgG(Fc)-HRP (1:70,000, A0170, Sigma). Three-hundred-eighty-four-well liquid handling was performed with a Precision XS microplate sample processor (BioTek), EL406 washer dispenser (BioTek), and BioStack Microplate stacker (BioTek). OD450 nm-620 nm was measured in an ELISA plate reader (BioTek Instruments, Epoch) and its software Gen5 version 3.03 was used to calculate EC_50_ values, further expressed as relative potency towards an internal calibrant for which the Binding Antibody Unit (BAU) was calculated using the WHO International Standard 20/136 in relation to the original Wuhan strain RBD. The graphics were created by GraphPad Prism 9.1. Significance was calculated by pairwise non-parametric multiple comparison ANOVA (Friedman’s test) with Dunn’s multiple comparisons test, using the Wuhan wt RBD values as the reference value for all three VOCs, but Omicron data were shown separately for better illustration.

### SARS-CoV-2 neutralization assays

SARS-CoV-2 strain G614 and VOCs (Delta and Omicron) were isolated from patients in Pavia and used for microneutralization assay [24, 25]. Briefly, 50 μL of the sample, starting from 1:10 in a serial twofold dilution series (up to 1:640), was added to two wells of a flat-bottom tissue-culture microtiter plate (COSTAR, Corning Incorporated), mixed with an equal volume of 100 Tissue Culture Infection Dose 50 (TCID50) of a SARS-CoV-2 strain, previously titrated and incubated at 33°C in 5% CO_2_. All dilutions were made in Eagle’s minimum essential medium with addition of 1% penicillin, streptomycin, and glutamine and 5 μg/mL of trypsin. After 1 h of incubation at 33 °C in 5% CO_2_, VERO E6 cells (VERO C1008 (Vero 76, cloneE6, Vero E6); ATCC® CRL-1586™) were added to each well. After 48 h of incubation at 33°C in 5% CO_2_, wells were stained with Gram’s crystal violet solution (Merck) plus 5% formaldehyde 40% m/v (Carlo ErbaSpA) for 30 min. Microtiter plates were then washed in running water. Wells were scored to evaluate the degree of cytopathic effect (CPE) compared with the virus control. Blue staining of wells indicated the presence of neutralizing antibodies. The neutralizing titer was defined as the maximum dilution giving a reduction of 90% of the CPE. The cut-off for positivity was ≥1:10. Positive and negative controls were included in all test runs.

## Results

### The Omicron RBD shows a slightly reduced binding to ACE2

**Fig. 1:**
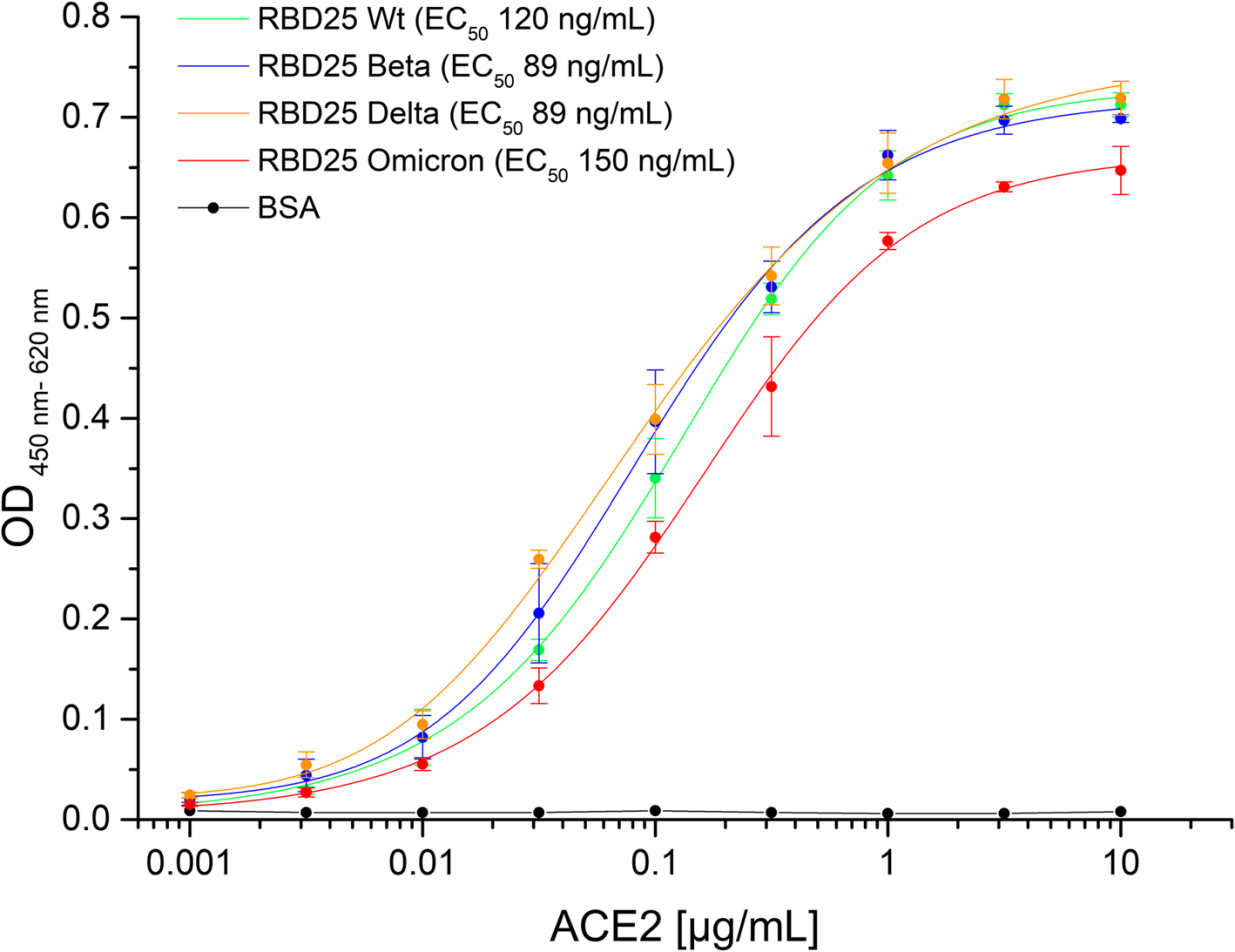
RBD variants binding to human ACE2. 300 ng/well immobilized Wuhan wt, Beta, Delta, or Omicron RBD were detected with human ACE2 (fusion protein with human Fc part) in titration ELISA. BSA was used as a negative control. Experiments were performed in triplicates and mean values are given. EC_50_ were calculated with OriginPro Version 9.1, fitting to a five-parameter logistic curve

The RBD of the original Wuhan strain, the Beta, Delta, and the Omicron variants were produced in insect cells and purified by IMAC and SEC. The quality of the recombinant proteins was analyzed by SDS-PAGE (Additional file 1: Fig. S1). All RBDs were immobilized on plates and binding of the soluble receptor ACE2 was analyzed by ELISA (Fig. 1). The Omicron RBD showed a slightly reduced binding to ACE2 (EC_50_ 150 ng/mL, respectively 5.6 nM) compared to the Wuhan strain RBD (EC_50_ 120 ng/mL, 4.6 nM). In contrast, an increased binding to Beta (EC_50_ 89 ng/mL, 3.4 nM) and Delta RBD (EC_50_ 89 ng/mL, 3.4 nM) was measured in comparison to the Wuhan strain. The affinities were also determined by microscale thermophoresis (MST) (Table 3, Additional file 1: Fig. S2). Again, the measured affinity for the Omicron RBD was slightly lower compared to Beta and Delta.

**Table 3:** RBD-ACE2 affinity measured by MST. All experiments were performed in titration in triplicates and analyzed by the MO Affinity Analysis software (NanoTemper) by Hill fit

***RBD***	***EC***_***50***_ ***(nM)***	***EC***_***50***_ ***confidence (nM)***
Wuhan strain	40.7	1.8
Beta	35.2	1.7
Delta	33.6	2.2
Omicron	42.3	1.6

### Human sera of COVID-19 patients and vaccinated persons show a reduced binding to Omicron RBD

**Fig. 2:**
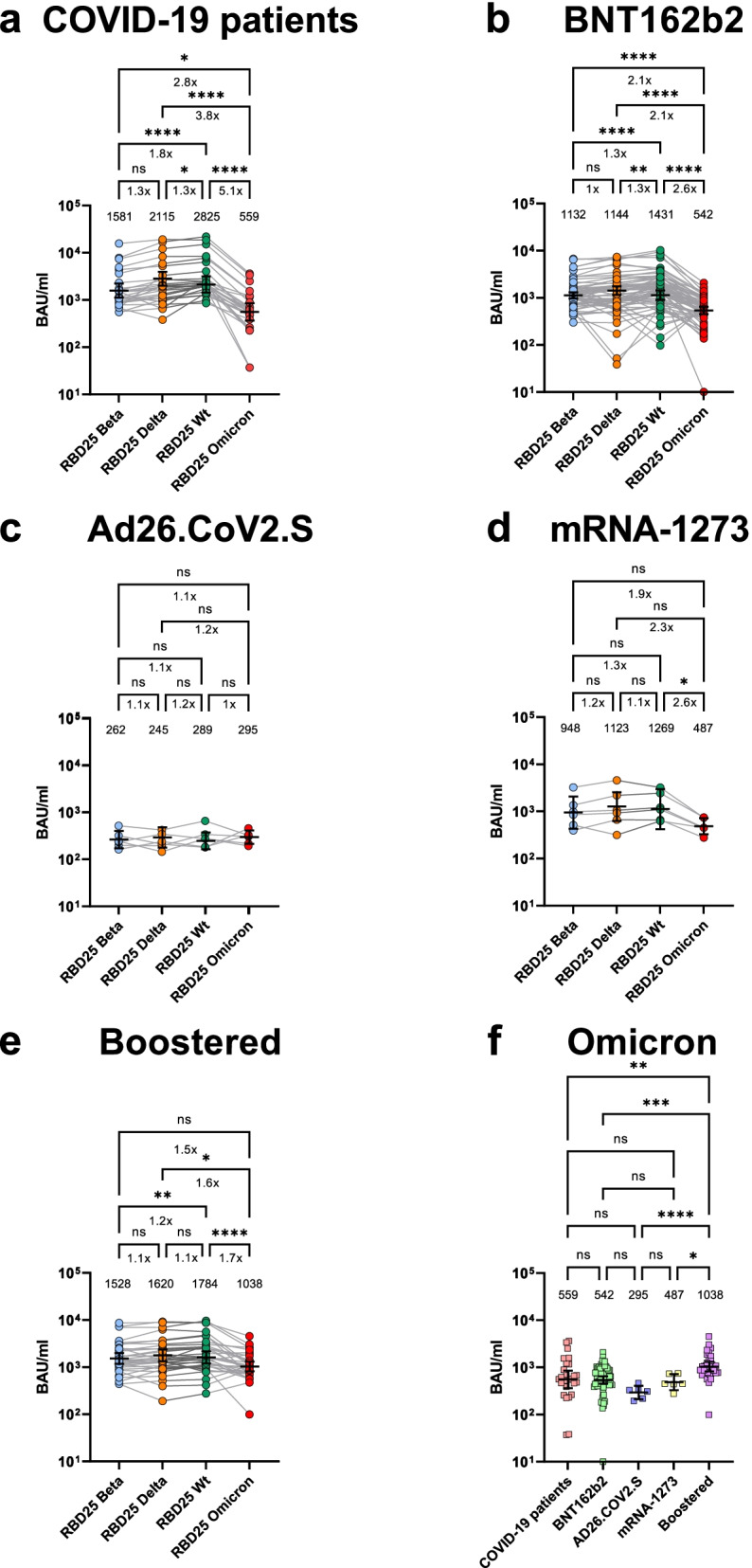
Human serum binding to SARS-CoV-2 Wuhan original strain, Beta, Delta, and Omicron RBD. **A** ELISA using sera from hospitalized COVID-19 patients. **B** ELISA using sera from 2×BNT162b2-vaccinated persons (7–52 days after 2nd immunization). **C** ELISA using sera from 1×Ad26.COV2.S-vaccinated (14–33 days 1st immunization). **D** ELISA using sera from 2×mRNA-1273 (5–55 days after 2nd immunization). **E** ELISA using sera from 2×BNT162b2 or 1× Ad26.COV2.S vaccinated + boosted with BNT162b2 or mRNA-1273 (5–49 days after 3rd or in case of Ad26.COV2.S 2nd immunization) binding to the Omicron variant. **F** Rearranged representation of the data presented in **A**–**E**. The ELISAs were performed as single-point titrations. The software Gen5 version 3.03 was used to calculate EC_50_ values, further expressed as relative potency in respect to an internal calibrant, for which the Binding Antibody Unit (BAU) was calculated using the WHO International Standard 20/136 titrated on Wuhan wt as reference. The geometrical mean values and the 95% CI are given in the graphs. The graphics and statistical analysis were performed with Graphpad Prism 9.1. For **A**–**E**: Friedman test with Dunn’s multiple comparisons test was performed on the four conditions per graph (WT, Beta, Delta, Omicron). For **F**: Kruskal-Wallis test with Dunn’s multiple comparisons test was performed. Geometric mean and 95% confidence interval are represented by error bars. Multiplicity adjusted *P* values are shown as follows: ns: *P* > 0.05, *P ≤ 0.05, **P ≤ 0.01, ***P ≤ 0.001, ****P ≤ 0.0001

The binding of human sera from hospitalized COVID-19 patients (Fig. 2A), from people vaccinated 2× with BNT162b2 (Corminaty) (7–43 days after second immunization) (Fig. 2B), once with Ad26.COV2.S (Janssen COVID-19 Vaccine) (14–33 days after immunization) (Fig. 2C), 2× with mRNA1273 (Spikevax) (5–55 days after second immunization) (Fig. 2D), or from mRNA vaccine boost-recipient vaccinees (5–49 days after boost vaccination, first immunization 2×BNT162b2 or 1×Ad26.COV2.S) (Fig. 2E) was analyzed by ELISA on Wuhan, Delta, Beta, and Omicron RBD. A direct comparison of the binding to Omicron RBD of all five serum groups is given in Fig. 2F.

The sera of COVID-19 patients (Fig. 2A), 2× BNT162b2 (Fig. 2B), and 2× mRNA-1273 vaccinees (Fig. 2D) showed a highly significant reduction in binding to Omicron RBD compared to Wuhan RBD in a non-parametric pairwise analysis. This reduction was more pronounced than the one observed against Beta or Delta RBD binding assays. Ad26.COV2.S group (Fig. 2C) showed in general a very low binding to all RBDs tested, suggesting a clearly weak immunogenicity of this vaccine formulation or posology.

Boost recipients had still a lower serum antibody binding to Omicron RBD compared to Wuhan, Beta, and Delta (Fig. 2E). However, the boost increased significantly the amount of anti-Omicron RBD antibodies in comparison to both the sera of vaccinees and COVID-19 patients (Fig. 2F). Interestingly, no significant difference was observed for people vaccinated first with Ad26.COV2.S and subsequently boostered with BTN162b2 in comparison to persons 3× vaccinated with a mRNA vaccine (Additional file 1: Fig. S3).

### Sera from vaccinated subjects show drastically reduced neutralization of authentic Omicron SARS-CoV-2 virus

**Fig. 3:**
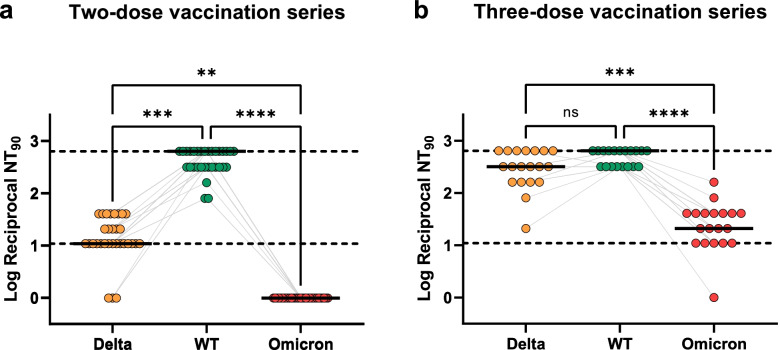
Neutralization of SARS-CoV-2 wild type (D614G), Delta, and Omicron. Neutralization of authentic SARS-CoV-2 wt (including D614G mutation), Delta, and Omicron using sera of 2× BNT162b-vaccinated (A) and BNT162b2 boost-vaccinated (B) individuals. Delta, WT, and Omicron 90% neutralization titers (NT90) and median of values are shown from healthcare workers that underwent two-dose vaccination series (**a**) and three-dose vaccination series (**b**). Samples were collected 2 weeks and 3 weeks after the last dose, respectively. Reciprocal titers were log10 converted (1 was added to all titers to allow undetectable neutralization to be plotted). Upper and lower dotted lines represent the upper and lower limit of detection (the equivalent of 1:640 and 1:10 titers, respectively). Gray lines represent matched samples from the same donor. Friedman test with Dunn’s multiple comparison was calculated and *P* values are shown in asterisks

The neutralization of authentic SARS-CoV-2 wt (with D614G mutation), Delta, and Omicron was analyzed using sera of 2× BNT162b-vaccinated and BNT162b2 boost-vaccinated individuals (Fig. 3). The neutralizing titer was defined as the maximum dilution giving a reduction of 90% of the cytopathic effect. The cut-off for positive neutralization was ≥1:10 serum dilution. For sera of the 2× BNT162b2-vaccinated persons, the neutralization titer of Delta was significantly reduced compared to wild type SARS-CoV-2. Even more clearly, no neutralization could be detected at all against Omicron for any serum sample. In contrast, sera of 3× BNT162b2 vaccinees showed no statistically significant difference in neutralization of both wt and Delta SARS-CoV-2 virus, highlighting the effective protection of BNT162b2 against Delta VOC. Despite a remarkable increase, neutralization titers against Omicron were still significantly reduced by 1–2 log fold compared to wild type SARS-CoV-2.

## Discussion

RBD-ACE2 interaction is a prerequisite for SARS-CoV-2 viral entry [8, 9, 26]. Surprisingly, the binding of the Omicron RBD to the ACE2 receptor appears to be reduced in our settings compared to the currently dominant Delta variant, both in an ELISA assay as well as by affinity measurement using MST. Both techniques led to different absolute values caused by the different technique principles [27] but resulted in the same trend. Still, the affinities measured by MST (40.7 nM for Wuhan RBD) were in the same range as the ACE2 affinities determined previously by surface plasmon resonance (44.2 nM) [28]. Several Omicron RBD mutations are assumed to increase the binding to ACE2: G339D, S477N, T478K, Q493K, and N501Y; others are proposed to be neutral: S371L, S373P, G446S, E484A, Q493R, and Q498R, or are assumed to reduce the binding to ACE2: S375F, K417N, G496S, and Y505H according to yeast display experiments performed by Starr et al. [26]. Hence, some bioinformatic models predicted an increase in the ACE2 binding affinity of Omicron RBD [29] while other models rejected this scenario [30, 31] and stated: “the Q493R/K mutations, in a combination with K417N and T478K, dramatically reduced the S1 RBD binding by over 100 folds” [30]. However, the latter considerations are all based on in silico modeling. Thus, RBD-ACE2 interaction involving a heavily mutated RBD, such as the one of Omicron VOC, may deviate from predictions and requires empirical biochemical testing. To our knowledge, this is the first comprehensive empirical analysis of Omicron RBD binding efficacy to the ACE2 receptor. According to our data, the binding of Omicron RBD to human ACE2 was not increased, but rather decreased, especially when compared to Beta and Delta. Despite the observed reduced ACE2 Omicron RBD interaction, ACE2 remains necessary for cell entry shown by a recent study with Omicron pseudotyped viruses [15]. An increased RBD ACE2 binding is leading to increased cell entry shown for Alpha, Beta, Gamma, and Delta [32]. For Omicron, it was shown that the cell binding is reduced (weaker cell-cell fusion activity) when comparing Omicron to Delta [33]. However, the decrease in RBD binding does not necessarily translate into reduced infectivity, as infectivity and replication are also defined by proteolytic spike processing, fusion efficacy, and RNA replication efficiency, just to name a few mechanisms [34–36]. Furthermore, the severity of disease depends on several factors, e.g., Delta and Beta show the same affinity to ACE2, but Delta leads to a more severe disease compared to Beta [37]. Nevertheless, our results argue that increased binding to the ACE2 receptors may be an unlikely cause of rapid Omicron spread. One has also to consider that we utilized in our work the originally available sequence with a Q493K mutation, whereas Q493R sequences have been published since. According to Starr et al. [26], the K mutation has an even higher affinity as the R mutation in in vitro binding studies. The here measured reduced binding of Omicron RBD to ACE2 was later confirmed by other studies [38, 39].

Importantly, RBD mutations may also lead to immune escape [40]. The humoral immune answer is a key factor for the antiviral defense [41] and the RBD is the main target of neutralizing antibodies [10, 11, 42]. RBD binding and neutralization capacity do correlate [43, 44]. The very low RBD binding in the Ad26.COV2.S compared to the COVID-19 patient group or the mRNA-vaccinated groups is in accordance with the former results [45] but impaired definite conclusions on Omicron immune escape upon Ad26.COV2.S vaccination. The reduced binding of sera from COVID-19 patients and mRNA vaccinees to the Omicron RBD was in accordance with our and other recent results [14–16, 46, 47] describing a highly reduced neutralization of the Omicron variant by human sera from vaccinated persons. However, a 2.6× reduction was observed in RBD binding while in this study the neutralization was under the detection limit for sera from 2× BNT162b2-vaccinated persons and other Omicron SARS-CoV-2 neutralization studies showing a reduction by a magnitude of 10× and more [14–16, 46, 47]. Therefore, the Omicron mutations mainly reduce the SARS-CoV-2 neutralization but not the RBD binding in the same measure: This indicates an immune escape focused on serum neutralization evasion. Besides neutralization escape, the reduced affinity seems to be compensated by increased viral replication shown in ex vivo explant cultures of human bronchus [48]. Sera from boost vaccine recipients showed a significant reduction in serum titers as well as 1–2 log fold reduced neutralization of the Omicron variant, whereas the titers and neutralization of the Delta variant did not significantly differ from the wild type. While we have measured a significant decrease of serum binding to Omicron RBD, the boost recipients still had a higher anti-Omicron RBD titer as well as at least a remaining neutralization activity compared to 2× vaccinated individuals. The efficacy of boost immunization in neutralization assays was also observed in other studies [14–16]. The timepoint of sampling (duration from vaccination, respectively infection, to sampling) can influence the antibody response. In this study, we focused on a timepoint after vaccination/infection where high antibody titers are expected. Prospective studies with boosted individuals will be pursued in the near future when the availability of subjects with longer time spans after the third dose will be available.

The results of this Omicron RBD study are a snapshot of the current situation. According to the sequencing data deposited as GISAID (https://www.gisaid.org/) and the analysis on Outbreak.info [49], the frequency of the 15 aa mutations in the RBD is very dynamic, e.g., K417N, described for the initial Omicron variant to occur in ~35% (status 2021-12-14, 2146 sequences) of all sequenced Omicron isolates, is now retrieved in above 55% of sequenced viruses (status 2022-02-07, 873.492 sequences). S477N, T478K, and E484A were initially at ~47% (status 2021-12-14, 2146 sequences), now instead above 88%, as N501Y (status 2022-02-07, 873.492 sequences). The K417N mutation is a key mutation also in the Beta variant, the T478K mutation instead in the Delta variant, and N501Y in the Alpha, Beta, and Gamma variants [50]. All of these mutations may contribute to both ACE2 binding efficacy and immune escape. Therefore, Omicron variants with alternative mutations might evolve in the near future and alter the antibody recognition and/or the ACE2 binding efficacy. More comprehensive studies of various subvariants in the Omicron family may shed light on their biochemical and immunological properties and understand the potential for future SARS-CoV-2 evolution.

## Conclusions

The Omicron RBD had a lower affinity to ACE2 compared to Beta and Delta, arguing that improved ACE2 binding is not a likely driver of Omicron evolution. Serum antibody titers from COVID-19 patients or mRNA vaccinees were significantly lower against Omicron RBD compared to the original Wuhan strain. Neutralization of Omicron SARS-CoV-2 by the serum of BNT162b2 vaccinees was completely diminished, indicating an immune escape focused on neutralizing antibodies. Nevertheless, a boost vaccination increased the level of anti-RBD antibodies against Omicron, and neutralization of authentic Omicron SARS-CoV-2 was at least partially restored.

## Supplementary Information

**Additional file 1: Fig. S1.** SDS-PAGE of the RBD25 variants. 2 μg of the indicated purified RBD25 variant in Laemmli buffer containing 5% beta-mercaptoethanol were heated to 95°C for 10 min and run on 15% SDS-PAGE. **Fig. S2.** MST measurements of the ACE2-hFc RBD interaction. Triplicates were measured and Hill fit was applied. **Fig. S3.** Human serum from boost vaccinated persons binding to SARS-CoV-2 Wuhan original strain, Beta, Delta and Omicron RBD. ELISA using sera from persons vaccinated first with Ad26.COV2.S and then boostered with BTN162b2 or persons fully vaccinated with a mRNA vaccine and boostered also with a mRNA vaccine. Kruskal-Wallis test with Dunn’s multiple comparisons test was performed. Geometric mean and 95% confidence interval are represented by error bars.

## Data Availability

Further information and requests for resources and reagents should be directed to and will be fulfilled by the Lead Contact, Michael Hust (m.hust@tu-bs.de). This includes antibodies, plasmids, and proteins. All reagents are available on reasonable request after completion of a Material Transfer Agreement.

## Abbreviations

ACE2: Human angiotensin-converting enzyme-2
CPE: Cytopathic effect
DOL: Degree of labeling
EC50: Half-maximal effective concentration
IMAC: Immobilized metal affinity chromatography
MST: Microscale thermophoresis
RBD: Receptor binding domain
SEC: Size exclusion chromatography
TCID50: Tissue Culture Infection Dose 50
TMB: Tetramethylbenzidine
VOC: Variant of concern
