# Supplementary material for: Human serum from SARS-CoV-2-vaccinated and COVID-19 patients shows reduced binding to the RBD of SARS-CoV-2 Omicron variant
Source: BMC Med. 2022 Mar 3;20:102. doi: 10.1186/s12916-022-02312-5 (PMC8890955; doi:10.1186/s12916-022-02312-5)

Additional File 1: Supplementary Figures

Figure S1: SDS-PAGE of the RBD25 variants. 2 µg of the indicated purified RBD25 variant in Laemmli buffer containing 5 % beta-mercaptoethanol were heated to 95°C for 10 min and run on 15% SDS-PAGE.

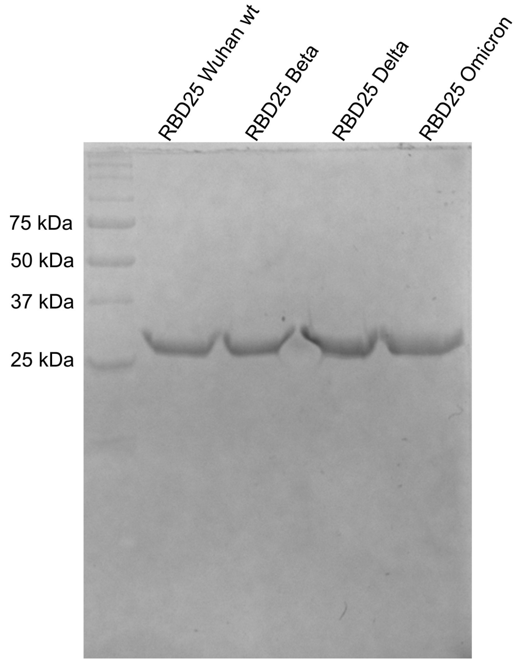

Figure S2: MST measurements of the ACE2-hFc RBD interaction. Triplicates were measured and Hill fit was applied.

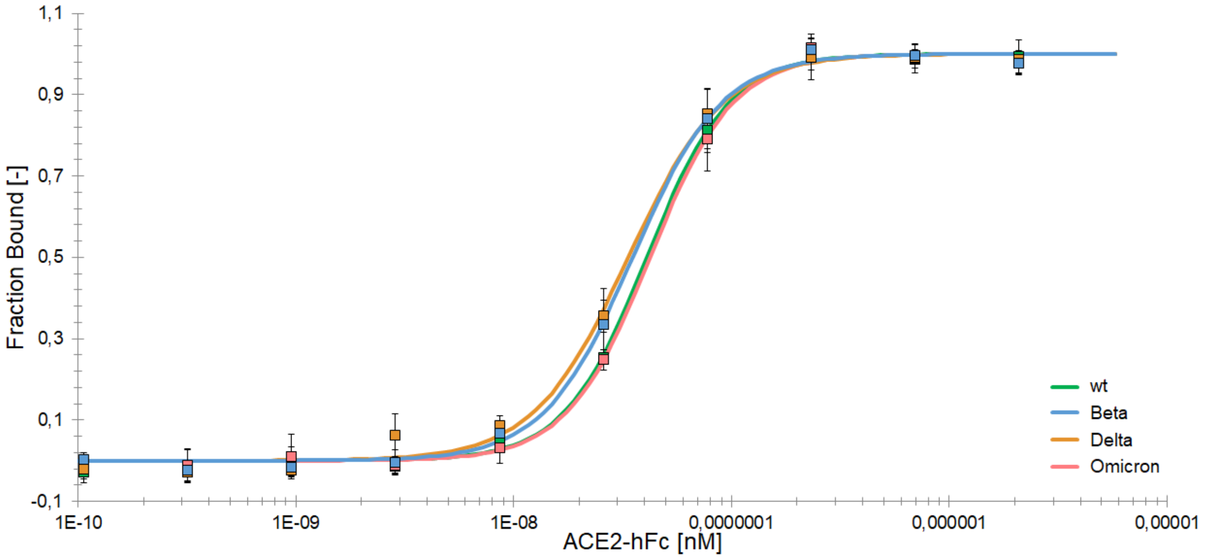

Figure S3: Human serum from boost vaccinated persons binding to SARS-CoV-2 Wuhan original strain, Beta, Delta and Omicron RBD. ELISA using sera from persons vaccinated first with Ad26.COV2.S and then boostered with BTN162b2 or persons fully vaccinated with a mRNA vaccine and boostered also with a mRNA vaccine. Kruskal-Wallis test with Dunn's multiple comparisons test was performed. Geometric mean and 95% confidence interval are represented by error bars.

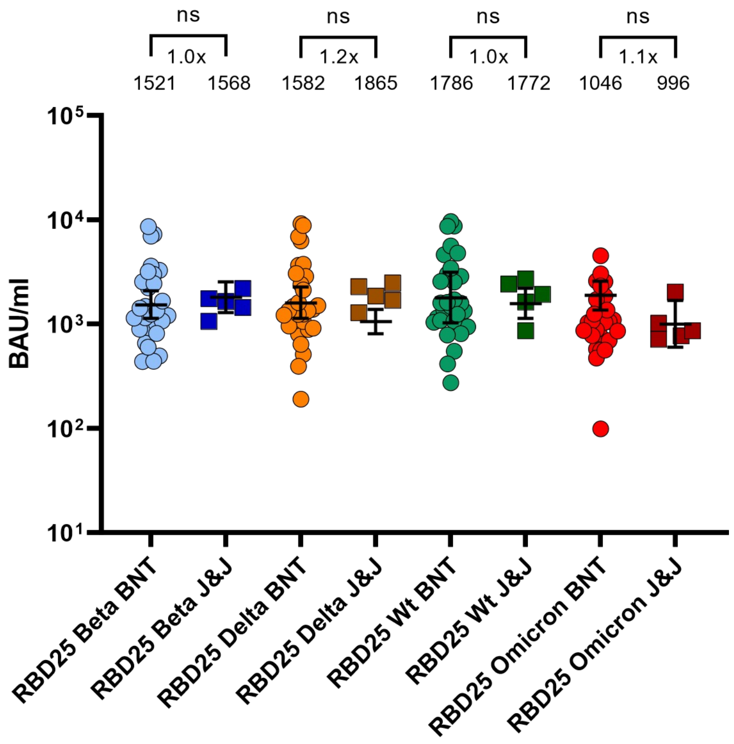

Supplement: Supplementary file 1 — Additional file 1: Fig. S1. SDS-PAGE of the RBD25 variants. 2 μg of the indicated purified RBD25 variant in Laemmli buffer containing 5% beta-mercaptoethanol were heated to 95°C for 10 min and run on 15% SDS-PAGE. Fig. S2. MST measurements of the ACE2-hFc RBD interaction. Triplicates were measured and Hill fit was applied. Fig. S3. Human serum from boost vaccinated persons binding to SARS-CoV-2 Wuhan original strain, Beta, Delta and Omicron RBD. ELISA using sera from persons vaccinated first with Ad26.COV2.S and then boostered with BTN162b2 or persons fully vaccinated with a mRNA vaccine and boostered also with a mRNA vaccine. Kruskal-Wallis test with Dunn’s multiple comparisons test was performed. Geometric mean and 95% confidence interval are represented by error bars. [file 12916_2022_2312_MOESM1_ESM.pdf]
